# Crevice Corrosion Behavior of Alloy 690 in High-Temperature Aerated Chloride Solution

**DOI:** 10.3390/ma15155434

**Published:** 2022-08-07

**Authors:** Fangqiang Ning, Jibo Tan, Ziyu Zhang, Xiang Wang, Xinqiang Wu, En-Hou Han, Wei Ke

**Affiliations:** 1 CAS Key Laboratory of Nuclear Materials and Safety Assessment, Liaoning Key Laboratory for Safety and Assessment Technique of Nuclear Materials, Institute of Metal Research, Chinese Academy of Sciences, Shenyang 110016, China; 2 School of Materials Science and Engineering, Shandong University of Science and Technology, Qingdao 266590, China

**Keywords:** Alloy 690, high-temperature corrosion, crevice corrosion, oxidation, chlorination

## Abstract

Crevice corrosion behavior of Alloy 690 in high-temperature aerated chloride solution was studied using a self-designed crevice device. The SEM, EDS, XRD, and XPS analyses results indicated that the oxide films outside the crevice consisted of Ni-Cr oxides containing a small amount of hydroxides, and the oxide films on crevice mouth consisted of a (Ni,Fe)(Fe,Cr)_2_O_4_ spinel oxides outer layer and a Cr(OH)_3_ inner layer, and the oxide films inside the crevice consisted of a α-CrOOH outer layer and a Cr(OH)_3_ inner layer. When crevice corrosion occurred, the hydrolysis of Cr^3+^ led to the formation of Cr(OH)_3_ inside the crevice, and caused the pH value of crevice solution to decrease, and Cl^−^ migrated from outside the crevice into inside the crevice due to electrical neutrality principle and accumulation. When the water chemistry inside the crevice reached the critical value of active dissolution of metal, the active dissolution of metal inside the crevice occurred. In addition, (Ni,Fe)(Fe,Cr)_2_O_4_ spinel oxides on the crevice mouth were formed by the deposition of metal ions migrated from inside the crevice. The mechanism of crevice corrosion and the formation mechanism of oxide films at different regions were also discussed.

## 1. Introduction

Nickel-based Alloy 690 is extensively used as steam generator (SG) tube material in nuclear power industries due to its high corrosion resistance [1,2,3,4,5]. However, Alloy 690 is vulnerable to crevice corrosion damage during long-time operation of pressurized water reactors (PWRs) [6,7,8]. Alloy 690 located in the crevices between the SG tube and tube support plate, and the tube sheet or deposits are easily attacked by corrosion [8,9,10,11,12,13,14]. The enrichment of aggressive impurity ions occurs inside the crevice during crevice corrosion. Thus, although the concentration of impurities in the SG feedwater is extremely low, and it can concentrate inside the crevice, producing an aggressive local environment and therefore accelerating corrosion degradation of SG tubes [14,15]. Thus, the crevice corrosion damage of SG tubes has become a key factor affecting the normal operation of PWRs.

Chloride ion (Cl^−^) is one of the most aggressive ions that can lead to localized corrosion of SG tube materials [10,16,17]. According to the crevice corrosion mechanism, Cl^−^ can migrate into the crevice and concentrate, while the hydrolysis of metal ions inside the crevice leads to a decrease in the pH value of the crevice solution [5,16], which can increase the corrosivity of crevice solution, therefore destroying the passive film of metal and causing crevice corrosion [18,19,20,21,22,23,24,25]. Many researchers have investigated the effects of Cl^−^ on the crevice corrosion behaviors of nickel-based alloys and stainless steels (SSs). Oldfield et al. [18] investigated the crevice corrosion behavior of 316 SS, and they found that the acidification of crevice solution was caused by the hydrolysis of Cr^3+^; this was due to the hydrolysis equilibrium constant of Cr^3+^ being smaller than other metal ions. Macdonald et al. [14] investigated the effects of Cl^−^ on crevice corrosion of Alloy 600 in 200 °C water and reported that Cl^−^ can increase the corrosion rate of Alloy 600 inside the crevice, and the crevice corrosion is not obvious in 200 °C water without Cl^−^. Nevertheless, little work has been carried out to investigate the crevice corrosion behavior of Alloy 690 in high-temperature chloride solutions. Our previous paper [13] investigated the crevice corrosion behaviors of Alloy 690 in 290 °C deaerated chloride solution and found that nodular corrosion occurred inside the crevice. The effect of dissolved oxygen (DO) on corrosion behaviors of nickel-based alloys in high-temperature water has been extensively studied [4,5]. The increase of DO concentration in high-temperature water can promote the corrosion of nickel-based alloys. In addition, M. Eškinja et al. [26] investigated the corrosion behavior of a ferritic steel in CO_2_ environment using the optimized linear polarization resistance method, and found that iron carbonate (FeCO_3_) was detected as the main component of the corrosion productions with highest protective performance on the surface of the ferritic steel.

The purpose of the present paper was to clarify the crevice corrosion mechanism of Alloy 690 in high-temperature aerated chloride solution and to find the effective paths to resist crevice corrosion in high-temperature pressurized water, thereby ensuring the safe service of SG tubes in PWRs. In this study, the crevice corrosion behavior of Alloy 690 in high-temperature aerated chloride solution was studied using a self-designed crevice device. The oxide films formed at different regions of the crevice sample of Alloy 690 were characterized and the related mechanism of crevice corrosion was also discussed.

## 2. Experimental Section

### 2.1. Materials and Crevice Device

**Figure 1:**
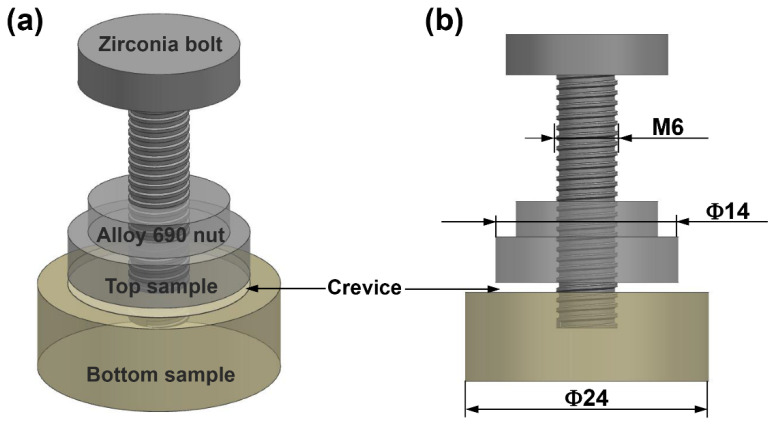
Schematic diagrams of (**a**) crevice corrosion device and (**b**) size of the crevice sample [5,6].

Table 1 shows the chemical composition of Alloy 690 used in the present paper. All crevice samples were gradually abraded to #2000 using emery paper followed by ethanol degreasing. Figure 1 shows the schematic diagram of crevice device that consisted of two Alloy 690 samples: a zirconia bolt and a Alloy 690 nut [5,6]. The use of the crevice apparatus has been described in detail in previous papers [5,6,7,27,28]. The crevice length and crevice width were controlled at 4 mm and 125 μm respectively in the present paper, as shown in Figure 1b.

### 2.2. Crevice Corrosion Immersion Test

Crevice corrosion immersion test was carried out in a static autoclave made of Hastelloy C276. In fact, the concentration of Cl^−^ in bulk solution ranges from a few ppb to dozens of ppb in PWR secondary circuit, while it would reach hundreds of ppm inside the crevice [6,8,10,15]. In order to accelerate the test, the experimental solution was 0.0002 M (7.1 ppm) sodium chloride (NaCl) solution, which was prepared with deionized water (0.06 μs/cm conductivity) and was aerated. The crevice corrosion immersion test was carried out in 290 °C NaCl solution for 200 h.

### 2.3. Methodology

After the immersion test, the sample was cleaned carefully with alcohol and dried by hair dryer. The surface appearance of the crevice sample was examined using a Leica S6D stereomicroscope. The micro-morphologies of oxide films were characterized using a scanning electron microscope (FEI XL30 SEM), which was equipped with an Energy Dispersive Spectrometer (EDS). The structures of oxide films were analyzed using a D/Max 2400 X-ray diffraction (XRD) analyzer with Co K alpha radiation (λ = 1.78892 Å). The chemical compositions of oxide films were analyzed by an X-ray photoelectron spectroscopy (ESCALAB 250 XPS). The XPS sputtering area was 2 × 2 mm, and the spectrum was acquired on a 0.5 mm diameter spot [5,29,30,31]. More information about the XPS device and processing XPS data has been detailed in previous work [5,29,30,31].

## 3. Results

### 3.1. Surface Appearance

**Figure 2:**
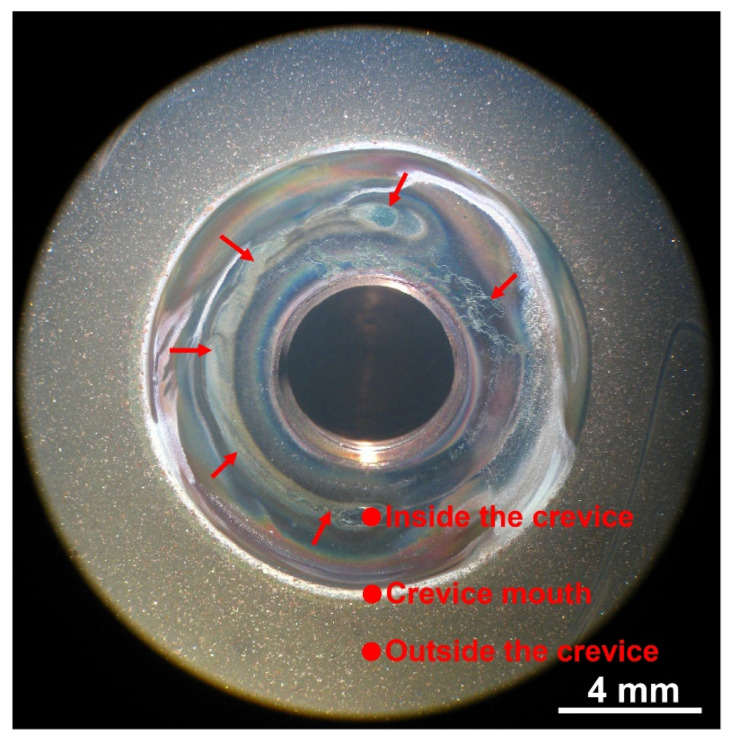
Surface appearance of the crevice sample exposed to 290 °C NaCl solution.

Figure 2 shows the surface appearance of the crevice sample exposed to 290 °C NaCl solution. It was found that active dissolution of the metal occurred inside the crevice (the region indicated by the red arrows), but not outside the crevice. The color and appearance of the oxide films inside and outside the crevice are different, indicating that the crevice corrosion did occur during the immersion test. According to the color of the oxide films, the sample surface was divided into three regions, namely, outside the crevice, crevice mouth, and inside the crevice.

**Figure 3:**
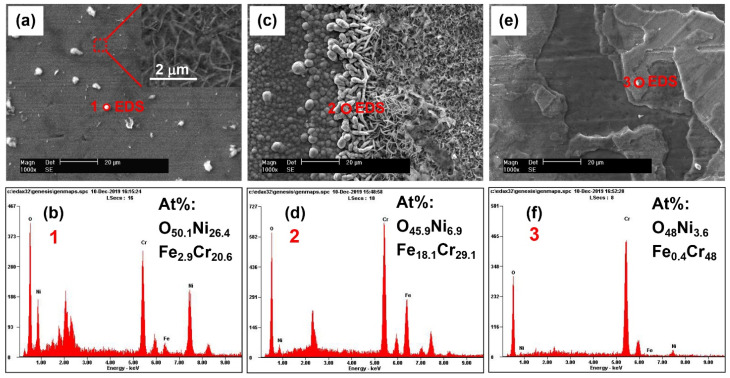
Surface morphology and EDS results of the oxide films formed at different regions of the crevice sample exposed to 290 °C NaCl solution: (**a**,**b**) outside the crevice, (**c**,**d**) crevice mouth, (**e**,**f**) inside the crevice.

Figure 3 shows the surface morphologies and EDS results of the oxide films formed at different regions of the crevice sample exposed to 290 °C NaCl solution. Although the EDS analysis is a semi-quantitative method, its results can reflect the relative content of various elements in the oxide films. Some oxide clusters deposited on the needle-like and flaky oxides were observed in the region outside the crevice (Figure 3a), and the EDS result (Figure 3b) indicates that these oxide clusters are rich in Ni and Cr. A large amount of vermicular and spherical oxides were densely distributed at crevice mouth (Figure 3c), and the EDS result (Figure 3d) indicated that these oxides are rich in Cr and Fe. In addition, it can be found the oxides formed at the crevice mouth were the most. A large amount of traces of active dissolution of metal was observed in the region inside the crevice (Figure 3e), and the EDS result (Figure 3d) indicated that these oxides are rich in Cr.

### 3.2. XRD Analysis of Oxide Films

**Figure 4:**
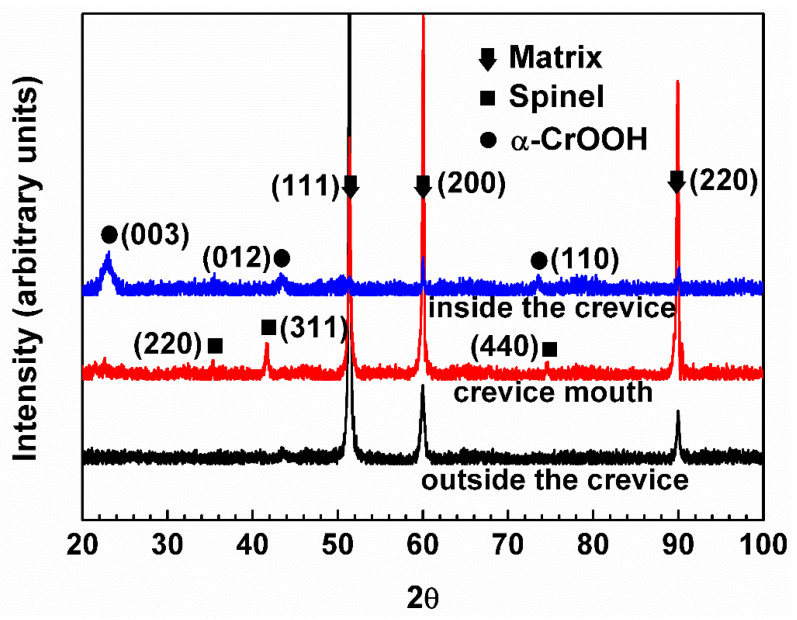
XRD analysis results of the oxide films at different regions of crevice sample exposed to 290 °C NaCl solution.

Figure 4 shows the XRD analysis results of the oxide films at different regions of crevice sample exposed to 290 °C NaCl solution. No characteristic peaks of oxides were identified outside the crevice, which may be because the oxide films were very thin. The characteristic peaks of spinel oxides were identified at crevice mouth and the EDS result suggests that the spinel oxides were rich in Fe and Cr, which indicates that the oxide films are mainly Fe-Cr spinel with a little Ni ((Ni,Fe)(Fe,Cr)_2_O_4_). Only the characteristic peaks of α-CrOOH with the crystal structure of rhombohedral [32] were identified inside the crevice.

### 3.3. XPS Analysis of Oxide Films

**Figure 5:**
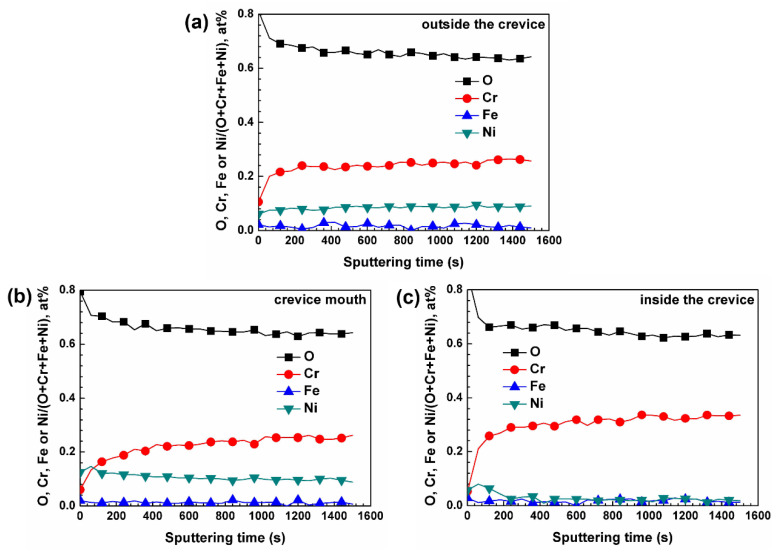
XPS depth profiles of the oxide films formed at different regions of crevice sample exposed to 290 °C NaCl solution: (**a**) outside the crevice, (**b**) crevice mouth, (**c**) inside the crevice.

Figure 5 shows the XPS depth profiles of the oxide films formed at different regions of crevice sample in 290 °C NaCl solution. It should be noted that there was no sputtering into substrate in the three regions. The oxide films formed outside the crevice and on crevice mouth were rich in Ni and Cr (Figure 5a,b), but the Cr content was higher than Ni. The oxide films formed inside the crevice were rich in Cr (Figure 5c). However, the oxide films formed on crevice mouth were mainly Fe-Cr spinel (EDS, XRD), which is inconsistent with the XPS analysis. This may be because the width of the Fe-Cr spinel layer formed on crevice mouth is very small, so the oxide films formed outside and inside the crevice (Figure 5c) were also sputtered during XPS sputtering.

**Figure 6:**
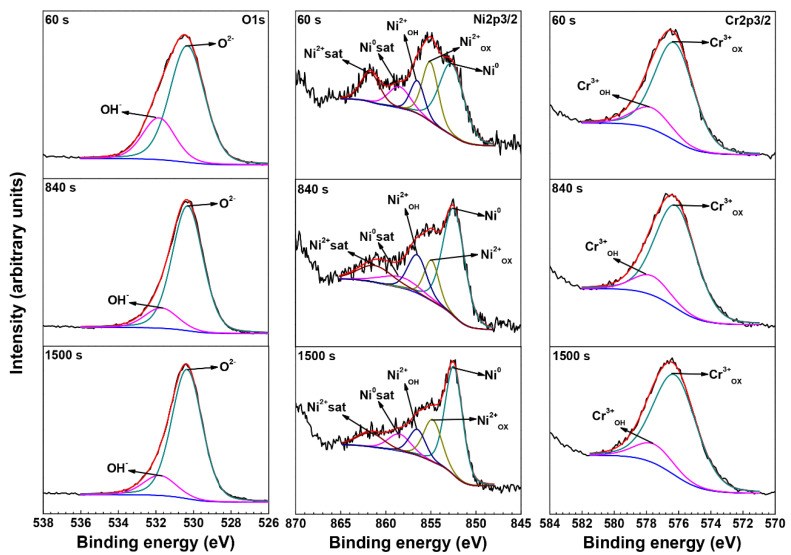
The O 1s, Ni 2p3/2 and Cr 2p3/2 core lever spectra outside the crevice of the crevice sample exposed to 290 °C NaCl solution.

Figure 6 shows the O 1s, Ni 2p3/2 and Cr 2p3/2 core lever spectra outside the crevice of the crevice sample exposed to 290 °C NaCl solution. Because there is a large amount of adsorbed oxygen and carbon on the outmost surface, the data were distorted at 0 s sputtering [5]. The sputtering time was 60 s, 840 s, and 1500 s. Table 2 shows the binding energy of targeted oxides or hydroxides [12,33,34,35,36,37,38,39,40]. The peak of O^2−^ dominated throughout the sputtering progress, indicating that the oxide films were mainly oxides. The peaks of Ni^2+^_OX_ and Cr^3+^_OX_ also dominated throughout the sputtering progress, and the peaks of Ni^2−^_OH_ and Cr^3+^_OH_ were very weak, indicating that the oxide films were mainly Ni-Cr oxides and contained a little Ni(OH)_2_ and Cr(OH)_3_. In addition, it could be found that the intensity of OH^-^ decreased and the intensity of O^2−^ increased with increasing of sputtering time, indicating that the hydroxides mainly exist in the outer layer of the oxide films, while the inner layer of the oxide films are mainly oxides.

**Figure 7:**
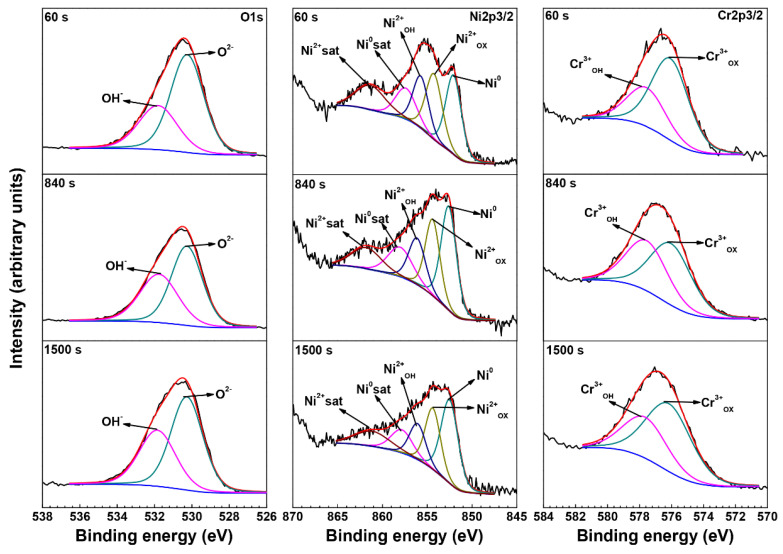
The O 1s, Ni 2p3/2 and Cr 2p3/2 core lever spectra on the crevice mouth of the crevice sample exposed to 290 °C NaCl solution.

Figure 7 shows the O 1s, Ni 2p3/2 and Cr 2p3/2 core lever spectra on crevice mouth of the crevice sample exposed to 290 °C NaCl solution. The peak of O^2−^ dominated throughout the sputtering progress, indicating that the oxide films on crevice mouth were also mainly Ni-Cr oxides. However, the intensities of OH^−^ and Cr^3+^_OH_ peaks on the crevice mouth were stronger than outside the crevice, indicating the content of Cr(OH)_3_ formed on crevice mouth was greater than that outside the crevice. In addition, XRD and EDS analyses indicated that the oxide films contain Fe-Cr spinel. Thus, the oxide films formed on crevice mouth consist of Ni-Cr oxides and Fe-Cr spinel and contain some Cr(OH)_3_.

**Figure 8:**
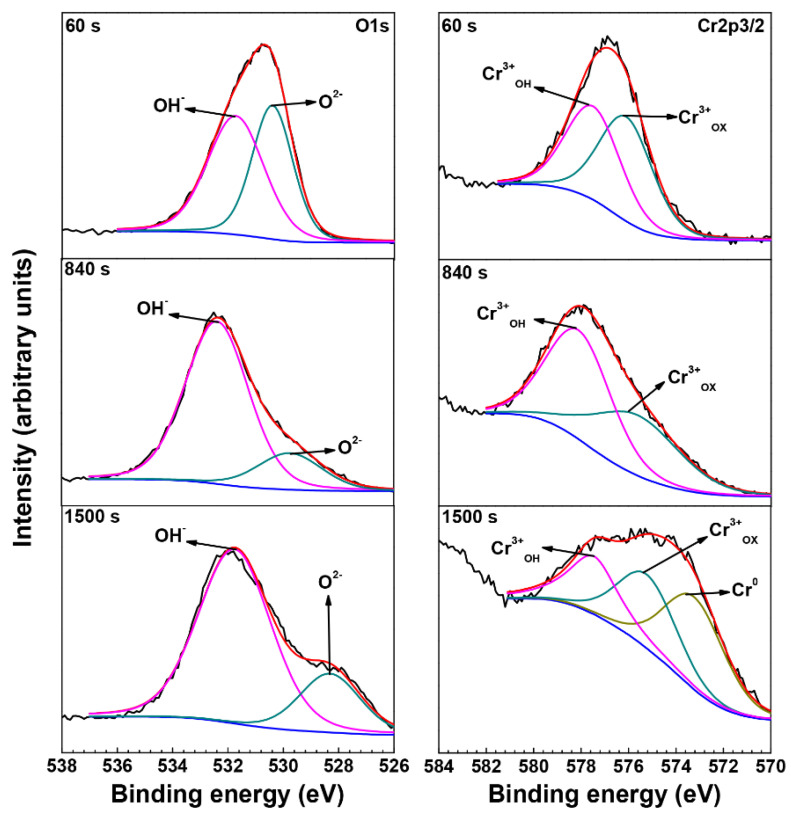
The O 1s and Cr 2p3/2 core lever spectra inside the crevice of the crevice sample exposed to 290 °C NaCl solution.

Figure 8 shows the O 1s and Cr 2p3/2 core lever spectra inside the crevice of the crevice sample exposed to 290 °C NaCl solution. The intensity ratio of O^2−^ to OH^−^ and the intensity ratio of Cr^3+^_OX_ to Cr^3+^_OH_ were approximately 1 at 60 s sputtering, indicating that the oxide films on the outmost surface inside the crevice are α-CrOOH, which is consistent with the XRD analysis. The peak of OH^−^ dominated with the sputtering time increasing to 840 s and 1500 s, indicating the oxides beneath α-CrOOH in the oxide films were mainly Cr(OH)_3_. Thus, the oxide films formed inside the crevice consist of a α-CrOOH outer layer and a Cr(OH)_3_ inner layer.

## 4. Discussion

The SEM, EDS, XRD, and XPS analyses results indicate that crevice corrosion did occur in Alloy 690 during the immersion test and the corrosion mechanisms at different regions of the crevice sample are different. In our previous paper, the oxide films formed outside of Alloy 690 crevice sample in 290 °C pure water containing 3 ppm DO consisted of an Ni-Fe spinel outer layer and a porous NiO inner layer [5,7]. This is because Cr-containing oxides in the oxide films are thermodynamically unstable in oxygenated high-temperature water and dissolve, resulting in the formation of the porous NiO layer [41,42,43,44]. However, the oxide films outside the crevice were Cr-rich oxides in the present paper. Many researchers have reported that the mechanism of Cl^−^ promoting metal corrosion is that it can promote the dissolution of Ni and Fe in the metal [10,13,17]. The dissolution rate of Ni and Fe induced by Cl^−^ may be higher than that of Cr induced by high DO concentration, resulting in the oxide films being rich in Cr. The formation mechanism of the oxide films outside the crevice is that the preferential dissolution of Ni and Fe induced by Cl^−^ results in the formation of Cr-rich oxides at initial stage of corrosion. With increasing the immersion time, the Cr-rich oxides layer thickens, and a large amount of dissolved Ni^2+^ induced by Cl^−^ reacts with the Cr-rich oxides to form Ni-Cr oxides. In addition, a small amount of Ni^2+^ and Cr^3+^ hydrolyze to form hydroxides. Eventually, the oxide films outside the crevice consist of mainly Ni-Cr oxides and contain a little Cr(OH)_3_ and Ni(OH)_2_.

Metal ions are dissolved from substrate inside the crevice during crevice corrosion. These metal ions cannot quickly diffuse to outside the crevice due to the small crevice width, resulting in the enrichment of metal ions inside the crevice [19,45,46]. The hydrolysis of these metal ions can produce a mass of H^+^, causing the pH value of crevice solution to decrease. In order to maintain the electrical neutrality of crevice solution, Cl^−^ could migrate from outside the crevice into inside the crevice and accumulates, leading to the concentration of Cl^−^ being higher in crevice solution than bulk solution [21,47,48,49,50]. Thus, the corrosivity of crevice solution increases, promoting the corrosion of metal inside the crevice [21,47,48,49,50]. The crevice solution reaches the critical value of active dissolution of metal, which occurs inside the crevice [18,19]. In addition, the oxygen is depleted inside the crevice, resulting in the corrosion potential of metal being lower inside the crevice than outside the crevice. Thus, the metal inside the crevice acts as an anode while the metal outside the crevice acts as a cathode [18,19,21].

The hydrolysis constant of Cr^3+^ is smaller than Fe^2+^ and Ni^2+^, leading to the formation of Cr(OH)_3_ inside the crevice, which could be verified by the XPS results (Figure 8). With the development of crevice corrosion, the hydrolysis of Cr^3+^ produces a large amount of Cr(OH)_3_, which leads to the pH of crevice solution reaching the critical value of active dissolution of metal, and promotes the dissolution of metal inside the crevice as shown in Figure 2 and Figure 3e. However, XRD and XPS analyses (Figure 4 and Figure 8) indicate that α-CrOOH is present on the outmost surface inside the crevice. Kuang et al. [4] also reported that α-CrOOH was present on Alloy 690 in high-temperature water containing low DO. In the present paper, the α-CrOOH may be converted from Cr(OH)_3_. Thus, the oxide films inside the crevice consist of a α-CrOOH outer layer and a Cr(OH)_3_ inner layer.

The dissolved metal ions from substrate inside the crevice diffused gradually out of the crevice with the development of crevice corrosion. When these metal ions diffused to the crevice mouth, they deposited to form a large amount of (Ni,Fe)(Fe,Cr)_2_O_4_ spinel oxides (Figure 3 and Figure 4). This is due to the DO concentration on the crevice mouth being close to outside the crevice [20,28]. Thus, the oxides formed on the crevice mouth are the greatest. The structure of spinel oxides is face-centered cubic (FCC), and the spinel oxides (AB_2_O_4_) with space group Fd-3m involve two sites, crystallographically distinct: the tetrahedral site A and the octahedral site B [51]. In turn, these oxides formed on crevice mouth could enhance the blocking effect and result in severe crevice corrosion. In addition, the hydrolysis of Cr^3+^ migrated from the crevice also produced some Cr(OH)_3_ on the crevice mouth. Eventually, the oxide films on the crevice mouth consisted of a spinel oxides outer layer and a Cr(OH)_3_ inner layer.

**Figure 9:**
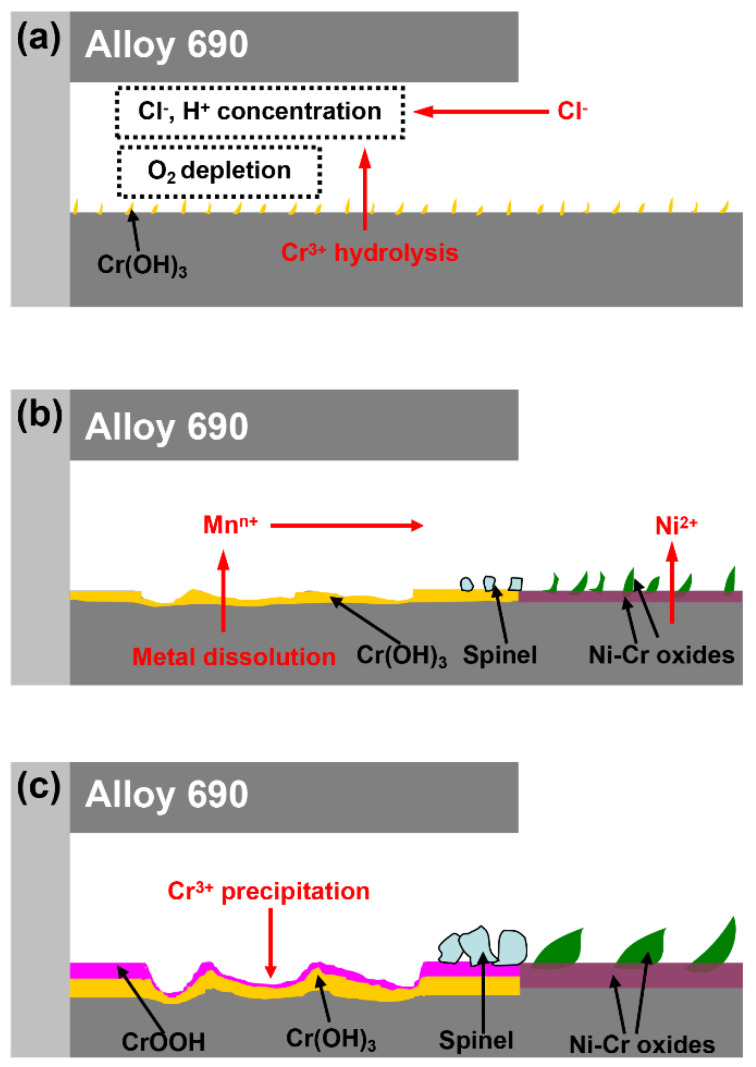
Schematic of crevice corrosion of Alloy 690 exposed to 290 °C NaCl solution. (**a**) initial stage of crevice corrosion (**b**) propagative stage of crevice corrosion (**c**) stable stage of crevice corrosion.

Figure 9 shows the schematic of crevice corrosion of Alloy 690 in 290 °C aerated chloride water. At the initial stage of crevice corrosion, the water chemistry inside and outside the crevice was consistent. The preferential dissolution of Fe and Ni induced by Cl^−^ resulted in the formation of Cr-rich oxides inside and outside the crevice. With the development of crevice corrosion, a large amount of metal ions dissolved from substrate accumulated inside the crevice, and the hydrolysis of Cr^3+^ caused the acidification of the crevice solution. Meanwhile, Cl^−^ migrates from outside the crevice into inside the crevice and accumulates due to electrical neutrality principle (Figure 9a). In addition, the oxygen is depleted inside the crevice because the oxygen transport is restricted by crevice geometry, resulting in the corrosion potential of the metal being lower inside the crevice than outside the crevice. Thus, the metal inside the crevice acted as an anode while the metal outside the crevice acted as a cathode (Figure 9a). When the water chemistry reached the critical value of active dissolution of metal, the active dissolution of metal occurred inside the crevice and the reduction of oxygen occurred outside the crevice (Figure 9b). In addition, the hydrolysis of Cr^3+^ inside the crevice resulted in the formation of Cr(OH)_3_. The dissolved metal ions induced by Cl^−^ inside the crevice diffused to the crevice mouth and deposited to form spinel oxides on the crevice mouth (Figure 9b). With the further development of crevice corrosion, a large amount of Cr(OH)_3_ was formed inside the crevice and a large amount of spinel oxides was formed on the crevice mouth (Figure 9c). In addition, Cr(OH)_3_ on the outmost surface inside the crevice was converted to α-CrOOH (Figure 9c). A large amount of deposited spinel oxides on the crevice mouth enhanced the blocking effect and resulted in severe crevice corrosion.

## 5. Conclusions

Crevice corrosion behavior of Alloy 690 in 290 °C aerated chloride solution was studied using a self-designed crevice device. The following conclusions can be drawn.

(1) The oxide film formed outside the crevice consisted of Ni-Cr oxides containing a small amount of Cr(OH)_3_ and Ni(OH)_2_, and the oxide film formed on the crevice mouth consisted of a (Ni,Fe)(Fe,Cr)_2_O_4_ spinel oxides outer layer and a Cr(OH)_3_ inner layer, and the oxide film formed inside the crevice consisted of a α-CrOOH outer layer and a Cr(OH)_3_ inner layer.

(2) The hydrolysis of Cr^3+^ led to the formation of Cr(OH)_3_ inside the crevice, and caused the pH value of crevice solution to decrease, and Cl^−^ migrated from outside the crevice into inside the crevice and accumulated due to the electrical neutrality principle. The water chemistry inside the crevice reached the critical value of active dissolution of metal with the development of crevice corrosion, thereby resulting in the active dissolution of metal inside the crevice.

(3) The metal ions dissolved from substrate diffused from inside the crevice to crevice mouth and deposited to form a large amount of (Ni,Fe)(Fe,Cr)_2_O_4_ spinel oxides, which enhances the blocking effect and result in a severe crevice corrosion.

## Figures and Tables

**Table 1:** Chemical compositions of Alloy 690 (wt. %).

Material	C	N	S	P	Mn	Ti	Al	Si	Cu	Fe	Cr	Ni
Alloy 690	0.03	0.013	0.001	0.007	0.293	0.202	0.202	0.292	0.01	10.61	30.04	58.3

**Table 2:** Binding energies of XPS peaks.

Element	Species	Binding Energy (eV)
O	O^2−^ (1s)	530.3
	OH^−^ (1s)	531.7
Ni	Ni^0^ (2p3/2)	852.7
	Ni^0^_sat_ (2p3/2)	858.5
	Ni^2+^_OX_ (2p3/2)	854.4
	Ni^2+^_OH_ (2p3/2)	856.5
	Ni^2+^_sat_ (2p3/2)	861.7
Cr	Cr^0^ (2p3/2)	574.3
	Cr^3+^_OX_ (2p3/2)	576.1
	Cr^3+^_OH_ (2p3/2)	577.6

## Data Availability

Not applicable.
